# Age-dependent differences in using FinTech products and services—Young customers versus other adults

**DOI:** 10.1371/journal.pone.0293470

**Published:** 2023-10-26

**Authors:** Dorota Krupa, Michał Buszko

**Affiliations:** Department of Financial Management, Nicolaus Copernicus University in Torun, Torun, Poland; Universiti Kebangsaan Malaysia, MALAYSIA

## Abstract

The purpose of this paper was to identify and evaluate differences in the attitudes to using FinTech products and services in Poland adopted by two study cohorts–one comprised of young customers, born no earlier than in 1990, and the other comprised of other adults. The main motivation for our research was to answer the question if young people growing up in the market economy will behave differently in the use of FinTech than older generations living in the former political and economic system. We also wanted to find the factors that determine the perception and willingness to use FinTech in the mentioned age groups. The data discussed in the paper were provided by the CAWI survey that was conducted in 2020 and covered a sample of 1,153 adult Poles. To achieve our goal, we used nonparametric statistical testing and the backward stepwise logistic regression models. The research demonstrated that young customers showed considerably more interest in all the aspects of the use of FinTech within the framework of our study than the other adults. Regarding the experience of using FinTech, such determinants as the male gender, the larger household in which a given respondent lives, and the possibility of making financial decisions independently exerted more impact on the young customers cohort than on the other adults. Irrespective of their opinion about FinTech, the persons under 30 years of age are more likely to use FinTech beyond average than the other adults whereas the persons over 30 years of age will do so only if they evaluate FinTech very well.

## 1. Introduction

Technological progress and the digitisation of business processes in the financial services industry as well as the restrictions imposed due to the COVID-19 pandemic prompt more and more clients to depart from traditional offers of financial products and services. Not only has the ongoing transformation of the financial sector already led to the creation of increasingly digitised business processes and models [1], but it has also generated new products and services. The products and services offered in the financial market are heavily influenced by the intensive development and implementation of information technologies, the application of which is generally referred to as FinTech. The FinTech creates specific ecosystem consisting of FinTech start-ups, technology developers, government, financial customers, and traditional financial institutions [2]. It is characterised by a considerable diversity of products, distribution channels, risk profiles, but primarily novel and innovative solutions.

The emergence of FinTech has its roots in the rapid growth and widespread adoption of internet, mobile and communications technologies (including social media). Both technology and the internet have the significant impact on the way people behave, interact and communicate with each other [3]. Clearly, the technology and the Internet also have a broad impact on financial markets, institutions, and transactions, stimulating the creation of new ideas, products, and infrastructure. They also determine the behaviour of customers of financial institutions. As the affection turned out to be so significant and vital, the merging of two mentioned spheres started to be considered as a specific area of economy and named as FinTech.

One of the cohorts which is especially susceptible to the use of technology and the Internet is young persons, including children, youths, and young adults. They can be identified as a group that is particularly open to various innovations [4]. When compared to the other adults, they are more active in social networks [5–7] and more technology-dependent, which translates into the use of technological innovations offered by the financial market. The acquisition of first experiences in the financial market–followed by the shaping of young persons’ financial behaviour–determines that youths and young adults become prospective customers of financial institutions. As young persons are perceived as the agents of change [8], it is essential to distinguish and evaluate them as a group while identifying the factors influencing financial decisions. The fact that young customers can use the products and services offered by both banking and non-banking institutions relates to the process of their financial inclusion described in the literature [9].

Due to the assumed higher absorptive capacity of young people (young adults) for innovative solutions compared to other adults, our study will include the comparison of the two cohorts mentioned in use of Fintech solutions [4]. The purpose of this paper is then to identify and evaluate the differences in the attitudes to using FinTech products and services between young customers and the other adults. Our study will be based on the survey conducted in Poland.

In our work we test the hypothesis that young adults are more likely to use FinTech products and services than the customers from the other age cohort.

To verify the hypothesis and realize the purpose of the paper, we examine:

- the susceptibility to use FinTech in two age cohorts,
- the factors that determine the use of FinTech offers in each age cohort (the experience with FinTech products and services),
- he factors that determine a high susceptibility to use the FinTech, i.e., the above-average use.

Our study is based on the background of the Polish market. The choice of Poland was motivated by two main factors. The first, was the opportunity to obtain data from a large and at the same time representative research sample thanks to the cooperation with an appropriate opinion research center in Poland. The second reason for choosing the Polish market as the country of study was the specificity of the market itself. Poland belongs to the region of Central and Eastern Europe (CEE), i.e., countries that changed their political and economic systems in the late 80s and early 90s of the twentieth century [10,11]. This means that a significant proportion of adults in the CEE region remember or grew up under different conditions than their peers in the Western countries. The study of FinTech, especially in the context of confronting group of young adults with a group of other adults, means that we are investigating not only generational but also civilizational changes. We consider it interesting to find out if whether people embedded in the market economy with an easy access to modern economic knowledge and higher inclination to information and communication technologies will behave differently in the scope of using FinTech than the older generations who grew up in the former political and economic system, who are less inclined to new, innovative solutions but more experienced in traditional finance. We also want to find out what factors determine the perception and willingness to use FinTech by the two cohorts mentioned, and whether these factors differ between them. By combining these two groups we contribute to the science by filling the gap of insufficient research related to evaluation of intergenerational differences in the use of FinTech products and services, including attitudes, experience, and susceptibility to use it above average. As the intergenerational comparison is based on the cohorts that grew up in two different political and economic systems, what is typical for CEE countries, we provide the field for further research, and identification of differences in the intergenerational use of FinTech in the Western countries.

Apart from the motivation presented above one may add that that the Polish FinTech sector itself is developing very rapidly. In the years 2018–2023, the number of FinTech entities registered in Poland increased from 167 to 360, and including foreign entities and entities operating for FinTech, to 417. This represents an increase of around 115% and 150% respectively. The largest group of FinTechs are entities offering payments. More than half of the FinTech in Poland was created after 2016, which proves the dynamic growth of the sector, reflected in the emergence of the 30 new entities per year [12].

The rest part of the paper is structured as follows: we present the theoretical background and literature review, describe the materials and statistical methods used, and characterize data and research process. The next section presents the results divided into the general susceptibility to use FinTech in various age cohorts, experience with FinTech products and services, and determinants of the susceptibility to use the FinTech offer beyond average. The paper ends with the discussion and conclusions, including the practical implications and limitations of the research.

## 2. Theoretical background and the literature review

The theoretical background for the operation and development of FinTech can be associated with the TAM (Technology Acceptance Model). According to the original Technology Acceptance Model (TAM), the customer’s intention to adopt a new technology depends on its perceived suitability and the ease of use [13,14]. The model has been repeatedly expanded and supplemented to, among others, the Combined Technology Acceptance Model and Theory of Planned Behaviour. In 2003 the model was unified (as the Unified Theory of Acceptance and Use of Technology–UTAUT) and in 2012 it was adapted to consumer conditions as the UTAUT2 model [15]. The framework of the TAM model and its modifications has been used inter alia to explain the adoption of new technologies in banking [16–18], but it also proved useful in explaining the FinTech phenomenon [19,20]. Some authors [21,22] emphasised that the uniqueness of Fintech vitally influences the difference in the TAM explanation of technology adoption in FinTech compared to other technology areas, such as e-commerce. The study of how users adopt FinTech services based on the TAM approach can be found mostly in the literature. However, as noted by Solarz and Swacha-Lech [20], apart from the TAM model, FinTech research is carried out by examining the impact of various demographic, social and economic variables [23,24], from the perspective of the perceived risk [24], or by combining selected variables from different approaches. Although the FinTech itself is not a new notion [2], Butor-Keler and Polasik [25] demonstrate that it is becoming more and more popular subject in the field of finance. Nonetheless, just defining FinTech is a challenging task. Expert literature assumes a threefold approach to the definition of the notion of FinTech: by subject, by object, and a combination of both [26,27]. Defining FinTech by subject refers to the implementation and use of digital technologies by banking and non-banking financial institutions [28], while defining it by object focuses on the sector of non-banking financial institutions relying on new technologies [29]. FinTechs are often technology-enabled innovative young companies concentrating on financial services [30]. For the purposes of this paper, FinTech will be defined by object as it refers to the innovative technologies used by non-banking institutions in finance. Research emphasises the considerable influence of FinTech on the financial services sector and describes it as breakthrough innovation [31]. Conducted by Liu et al. [32], a comprehensive analysis of the FinTech business model demonstrates that the main trends in FinTech-related studies include mobile payments, microfinancing, P2P lending, crowdfunding, and blockchain. Lee and Shin [2] identify fintech business models implemented by the FinTech start-ups such as payment, wealth management, crowdfunding, lending, capital market, and insurance services. Other authors underlined additionally foreign exchange and stock investments as well as foreign transfers [33–36].

The results of research into FinTech customers are also available. They indicate that the lack of trust to others and the dissatisfaction with the relations between the customer and the financial institution are the major reasons why individuals decide to change the financial institution or recognise FinTech as the main service provider [37]. Conducted by Jünger and Mietzner [38] study of German households demonstrated that the three basic aspects–trust, transparency, and financial education–exert a considerable influence on the decisions concerning the use of FinTech.

From the point of view of the subject of this article, is the issue of intergenerational differences in the FinTech research is important. The works on human capital theory published in the 1960s by Schultz [39,40] and Backer [41], can be treated as the theoretical foundation of research into financial behaviour and decisions in the intergenerational context. These works pointed out the key role of non-financial factors–that is knowledge and skills treated as human capital–while explaining income inequality among people. At the same time, the researchers emphasised the necessity of investing into this capital, for example through education, so that it could yield a higher rate of return. In subsequent years, the human capital theory was developed by such scholars as Delavande et al. [42], Houston [43], Lusardi and Mitchell [44,45], and Lusardi et al. [46,47]. Among others, the theory considered financial literacy as a component of the human capital and determined its influence on various stages of one’s life cycle. The financial literacy model includes elements like financial knowledge, skills, and the influence of family and peers [48] as well as the attitudes shaping one’s financial behaviour [49]. A complex financial concept [50], financial welfare was recognised to be the major result of financial literacy. FinTech due to its dual character, i.e., innovative technologies and finance creates the natural link between young people, financial literacy, and human capital.

Used throughout this paper, the term ‘young adults’ should be referred to the generational identity discussed in the expert literature. The very term ‘generation’ is ambiguous and can be understood differently in social sciences [51–54]. A ‘generation’ is defined as a distinctive group of persons at a similar age, living at a similar location, and shaped by similar events happening at a given time [55]. A generation usually partakes in similar historical and social events whose outcomes are relatively constant throughout its life [56]. Regarding the above, today it is possible to identify five generations that are typically classified as per the year of birth of their members, that is:

- Traditionalists (born in or before 1945),
- Baby Boomers (born between 1946 and 1964),
- Generation X (born between 1965 and1981),
- Generation Y–Millennials (born between 1982 and 2000),
- Generation Z (born after 2000).

This division is conventional, which means that while reviewing the research of various authors, particularly those representing different academic disciplines, one may come across slightly different age bracket definitions as well as different names of individual generations, especially the youngest ones [57–61]. Characteristic of a given country, the socio-cultural and historical contexts are also essential as they may result in limited comparability (or a lack thereof) of generations in the Western and the post-communist Central and Eastern European countries, including Poland [62,63]. Cwynar [62] stresses that the smallest differences in the way various countries define similar generations can be seen in the youngest generations, such as Millennials [64]. Owing to the Internet and social media, these generations are the most socially and culturally homogeneous and at the same time, as indicated in the further part of the work, they are the most often studied in the literature in the context of financial aspects.

It must be emphasised that 1989 was the breakthrough year of political transition in Poland [11]. The persons born after the transformation grew up and were raised in a completely different economic reality than the older generations. When compared to the older population, they were raised in a digital culture and grew up in similar conditions as their peers in the Western countries.

The review of the literature definitively shows a relatively small number of works related to the use of FinTech by different generations of the society both in the Western cultures as well as CEE countries, where the living conditions of people of different age groups have been different. Table 1 provides an overview of the core research on various aspects of the use of FinTech in the generational context, which allowed to identify the research gap covered by this paper.

**Table 1:** Review of the selected studies to identify a research gap.

Authors and publications	Research group and country of the study	Generations analyzed	Purpose of the study
			FinTech aspects in the analysis
[23] Carlin B, Olafsson A, Pagel M. Fintech Adoption across Generations: Financial Fitness in the Information Age.	13,838 users of the financial software for European banks and financial institutions, Iceland	Baby Boomers (1946–1964) Gen X (1965–1980) Millennials (1981–2000)	“*This paper analyzes how better access to financial information via new technology changes use of consumer credit and affects financial fitness*.”
			The authors examined whether the introduction of smartphone applications has influenced users’ personal financial management and whether consumers have increased their access to information in response to the availability of new technologies.
[38] Jünger M, Mietzner M. Banking Goes Digital: The Adoption of FinTech Services by German Households.	643 households, Germany	no distinction between generations	“*Germany is falling behind its peers in adopting new digital technologies and financial services offered by non-bank high-tech startups (e*.*g*., *FinTech)*. *Using survey data*, *we analyze which FinTech services households are likely to adopt*.”
			The authors identified the factors (trust, transparency, and financial expertise) that make consumers to switch at least part of their financial services from traditional financial institutions to financial technology startups.
[62] Cwynar A. Financial Literacy, Behaviour and Well Being of Millennials in Poland Compared to Previous Generations: The Insights from Three Large Scale Surveys.	three different samples (1,055 Facebook users, 1,067 individuals, 1000 spouses and cohabitants), Poland	Millennials (1982–2000), Gen X (1965–1981), Baby Boomers (1946–1964) Silent Generation (born 1945 and earlier)	“*The goal of the article is to compare Millennials to non-Millennials as a whole*, *as well as to other generations treated in isolation*, *to check whether Millennials diverge in terms of financial literacy*, *behavior and well-being*.”
			The author does not directly examine the use of FinTech products and services but points to the need for further research on intergenerational differences in the context of technological progress.
[20] Solarz M, Swacha-Lech M. Determinants of the Adoption of Innovative FinTech Services by Millennials.	1,236 individuals, Poland	Millennials (1980–1995)	“*This paper analyzes and evaluates the selected determinants of using the innovative FinTech services by Millennials in Poland*.”
			The authors conducted a comprehensive review of the determinants (factors influencing the decisions about choosing a financial institution, offered access channel, benefits related to a given financial institution, demographic, and economic determinants) of using innovative services offered by FinTech.
[65] Daragmeh A., Lentner C., Sági J. FinTech payments in the era of COVID-19: Factors influencing behavioral intentions of “Generation X” in Hungary to use mobile payment.	1,120 individuals, Hungary	Generation X	“*This study aims to evaluate factors that influence Hungarian Generation X’s behavioral intentions to use mobile payment services during the pandemic*.*”*
			They examined the effects of subjective norms, perceived ease of use, perceived usefulness and perceived COVID-19 risk on the behavioural intentions to adopt mobile payments.

Source: Authors’ own elaborations.

Despite the growing number of the papers dedicated to the FinTech in general and to different aspects of their functioning [20,38,65], there is still a gap in the context of comparing the younger generation (young adults) with other adults. The issue of age as differentiating factor in the financial services can be easily found in many papers, especially those indicating the decreasing acceptance of new technologies with age (especially those used in online or mobile banking) [17,65–69] or directly indicate a negative relationship between the age and the use of Fintech solutions [20]. Nonetheless, there are no studies that indicate what factors are behind it, in relation to FinTech technologies. Moreover, in the intergenerational sense the papers related to CEE countries are mainly devoted to comparing Millennials and Z generation [20,36], but there is still not sufficient investigation of FinTech in the context of comparing young adults and other adults brought up in a different socio-political context, as it was in Poland. Therefore, we identified a research gap in assessing the differences between generations growing up in two different political and economic systems in the use of FinTech products and services, including attitudes, experience, and susceptibility to use them in an above-average way.

As most of the financial products and services currently available on the market are becoming FinTech, and as traditional financial institutions (e.g., banks or insurance companies) more and more frequently takes over the FinTech outlook, the knowledge related to FinTech functioning in inter-generation aspects turns out particularly important. The knowledge that is developing in this field may help to support effective inclusion in the financial market not only young people but also other adults in terms of fast changes in the information and communication technologies. Moreover, it may also prevent exclusion of older people from the financial market in the emergence of the FinTech era. The knowledge in the inter-generation use of FinTech may identify determinants of acceptance and willingness to use on a daily basis FinTech by the young and other adults.

In this paper the term ‘young adults’ will denote the persons who were born no sooner than in 1990 and were not older than 30 in the year the survey was conducted (2020). Consequently, the young adults’ cohort includes Generation Z and younger Millennials. The term other adults refer to people over 30 years of age.

## 3. Materials and methods

The paper relies on a survey conducted by a professional opinion poll organisation in October and November 2020 among a sample of 1,153 Poles over 18 years of age. The sample was a quota sample, which ensured the compliance of its structure with the population structure. The CAWI (Computer-Assisted Web Interview) method was used to conduct the survey and the research results were processed with the use of the IBM SPSS Statistics 26 software. The basic characteristics of the respondents participating in the survey are presented in Table 2.

**Table 2:** General presentation of survey participants.

		Frequency		Percent
Gender	Woman	593		51.4
	Man	560		48.6
Age	18–24	107		9.3
	25–34	206		17.9
	35–44	222		19.3
	45–54	172		14.9
	55–65	211		18.3
	66+	235		20.4
Level of formal education	Primary	25		2.2
	Gymnasium	40		3.5
	Vocational	286		24.8
	Secondary	466		40.4
	Bachelor	92		8
	Master	225		19.5
	Ph. D.	19		1.6
Type of formal education	Economic	189		16.4
	Non-economic	964		83.6
Marital status	Single	331		28.7
	In relationship	799		69.3
	Refusal to answer	23		2
Household size	Single	138		12
	Couple	372		32.3
	Small family (3–4 persons)		472	40.9
	Medium family (5–6 persons)	146		12.7
	Large family (over 7 persons)	25		2.2
Professional status	Employed	556		48.2
	Business owner	12		1.0
	Farmer	14		1.2
	Pensioner	350		30.4
	Unemployed	49		4.2
	Student	57		4.9
	Other	115		10.1

Source: Authors’ own calculations.

For the purposes of statistical data analysis, we conducted a study of the normality of the distribution of random variables for two cohorts studied (up to and including 30 years–young adults and over 30 years–other adults). As the Kolomogarov-Smirnov test showed a lack of normality of the distributions, we decided to use non-parametric statistical tests and modelling methods to disregard the assumption of normality of the distribution. Due to the ordinal nature of the variables, we used the nonparametric Mann-Whitney U test and logistic regression. We performed the Mann-Whitney U test at α = 0.05 to determine the general susceptibility to use FinTech products and services by young customers (born in 1990 or later and not older than 30 in 2020) and the other adults (over 30 years old) and we used two backward stepwise logistic regression models at α = 0.1 to evaluate the experience of using FinTech and to identify the determinants of the high susceptibility to use the FinTech offers (above-average use).

## 4. Results

### 4.1. The general susceptibility to use FinTech in various age cohorts

At the first stage of the study, we intended to compare:

- the experience in using FinTech products and services,
- the general susceptibility to use a variety of products (in respect of payments, foreign exchange, lending (non-banking institutions), lending platforms, insurance, cryptocurrencies, stock investment) offered by FinTech entities,
- general evaluation of FinTech,
- the possibility of exclusive use of FinTechs.

The FinTech products and services analysed in our study were selected based on observations of the Polish market and their availability among FinTech entities in Poland. The selection we also based on FinTech types commonly described in the literature presented in the Introduction section of this paper. The comparison concerned young customers was contrasted with that of the other adults (over 30 years old). The cohort of young adults (young customers) comprised of 230 persons and the cohort of the other adults (other customers) that comprised of 923 persons.

The comparison based on nonparametric Mann-Whitney U test [70] and presented in Table 3. shows differences between the age cohorts in respect of having experience with FinTech, having interest and willingness to use seven types of FinTech products, general evaluation of FinTech and the potential for exclusive use of FinTech.

**Table 3:** A comparison of young customers with the other adults.

		N	Mean Rank	M-W U Test Asymp. Sig. (2-tailed)
Having experience in use of FinTechs	up to 30	230	629.59	**
	over 30	923	563.90	
Payments	up to 30	230	712.62	***
	over 30	923	543.2	
Foreign exchange	up to 30	230	688.47	***
	over 30	923	549.22	
Lending (non-banking institutions)	up to 30	230	613.26	*
	over 30	923	567.96	
Lending platforms	up to 30	230	607.05	
	over 30	923	569.51	
Insurance	up to 30	230	663.59	***
	over 30	923	555.42	
Cryptocurrencies	up to 30	230	692.63	***
	over 30	923	548.19	
Stock investment	up to 30	230	677.35	***
	over 30	923	551.99	
FinTechs general evaluation	up to 30	167	407.2	**
	over 30	565	354.47	
Possibility of exclusive use of FinTechs	up to 30	230	665.73	***
	over 30	923	554.89	

Significance levels

*** < 0.001

** <0.01

* <0.05.

Source: Authors’ own calculations.

All the differences between the young customers and the other adults surveyed in the study occurred to be statistically significant except for the loans granted through lending platforms. In all cases the mean rank value for the persons up to 30 years of age turned out to be higher than in the other adults’ cohort.

### 4.2. Experience with FinTech products and services

In the next step, we decided to find factors that influence decisions about using FinTech offers in each age cohort. In the group of young customers 163 persons admitted that they had experience of using FinTech, which accounted for 72.6% of the cohort, while in the group of persons older than 30 years of age 565 respondents had experience of using FinTech products or services (61.2% of the other adults’ cohort). The comparison of the factors influencing using/not using FinTech was conducted by means of a binary choice model, i.e., logistic regression (the logit model), used to monitor the choices made by individuals [71].

In our case, we used the backward stepwise estimate and the model described with Eq 1.


$$Logit\;P(Y=1|X)=\beta_{0}+\sum_{i=1}^{n}\beta_{i}∙X_{i}+\xi$$ (1)

In the model, the dependent variable assumed as 1 was the possession of experience of using FinTech products and services by the respondents, whereas lack of such experience was assumed as 0. Moreover, we selected 20 explanatory variables (factors) characterising the respondents in respect of gender (X1), place of residence (X2), type and level of education (X3), marital status (X4), household size (X5), use of selected banking products (X6-X12), evaluation of FinTech (X13), independence in making financial decisions (X14), professional status (X15), being a farmer (X16), being retired or pensioner (X17), being a business owner (X18), being employed (X19), being a student of primary or other school (X20).

The description of the model parameters for the young customers cohort is presented in Table 4.

**Table 4:** Experience in using FinTech products and services (young customers).

	*β*	S.E.	Exp(*β*)	95% C.I. Low.	95% C.I. Up.
Man (vs. woman)	0.601	0.344	1.824*	0.929	3.582
Use of bank account	-0.897	0.341	0.408**	0.209	0.795
Use of prepaid card	2.737	1.054	15.44**	1.957	121.843
Independent financial decisions	1.475	0.579	4.371**	1.405	13.593
Household size (2 persons) vs. (1 person)	0.523	0.996	1.687	0.24	11.879
Household size (3–4 persons)	1.034	0.903	2.812	0.479	16.507
Household size (5–6 persons)	1.992	0.966	7.334*	1.103	48.752
Household size (7 persons and more)	2.153	1.427	8.608	0.525	141.107
Constant	-0.425	0.942	0.654		

Significance levels

*** < 0.001

** <0.010

* <0.100.

p-value of the test of model coeff. <0.001, N = 225, α = 0.1, Cox and Snell R^2^ = 0.139, Nagelkerke R^2^ = 0.201, H-L Test = 0.318.

Source: Authors’ own calculations.

Among the variables five of them were statistically significant. Two variables were related to socio-demographic aspects while the remaining concerned economic and financial issues.

The estimate of the model parameters for the other adults (over 30 years of age) is presented in Table 5.

**Table 5:** Experience in using FinTech products and services (adults over 30 years of age).

	*β*	S.E.	Exp(*β*)	95% C.I. Low.	95% C.I. Up.
Man (vs. woman)	0.314	0.147	1.369*	1.027	1.825
Use of mobile banking	0.656	0.148	1.927***	1.441	2.575
Use of prepaid card	0.771	0.338	2.161*	1.115	4.187
Use of credit card	0.296	0.156	1.344*	0.99	1.824
Independent financial decisions	0.309	0.182	1.362*	0.953	1.947
Household size (2 persons) vs. (1 person)	0.343	0.257	1.41	0.852	2.333
Household size (3–4 persons)	0.829	0.27	2.291**	1.351	3.886
Household size (5–6 persons)	1.053	0.338	2.867**	1.477	5.565
Household size (7 and more)	1.751	0.694	5.758*	1.477	22.454
Employed	0.267	0.153	1.306*	0.967	1.763
Constant	-0.934	0.277	0.393**		

Significance levels

*** < 0.001

** <0.010

* <0.100.

p-value of the test of model coeff. <0.001, N = 905, α = 0.1, Cox and Snell R^2^ = 0.090, Nagelkerke R^2^ = 0.123, H-L Test = 0.315.

Source: Authors’ own calculations.

In case of the modelling of the experience in use of FinTech products and services among adults over 30 years of age, seven independent variables turned out to be statistically significant with three concerning socio-demographic aspects and the remaining four pertaining to economic and financial issues.

### 4.3. Determinants of the susceptibility to use the FinTech offer beyond average

The study concerning the use of FinTech products and services by young customers and the other adults was expanded to include the modelling of high susceptibility to use the FinTech or, in other words, the susceptibility to use FinTech offer beyond average. We decided that the above-average interest in the FinTech offer should be defined as more than a half of the value of the score obtained evaluations of declarations of using FinTech regarding seven aspects (payments, foreign exchange, lending (by non-banking institutions), lending platforms, insurance, cryptocurrencies, and stock investment). The respondent could evaluate each aspect of the declaration of using FinTech on the scale from 1 (definitely not) to 5 (definitely yes), which–with seven aspects evaluated–gave a minimum score of 7 and a maximum score of 35. The mean score for the seven segments was 21 and set the threshold for another division within the framework of which the persons from both the young customers cohort and the other adults’ cohort were divided into two sub-cohorts:

- showing above-average (high) interest in FinTech offers–this sub-cohort included the persons who scored over 21 points;
- showing average or less-than-average (low) interest in FinTech offers–this sub-cohort included the persons who scored 21 points or fewer.

Out of 163 young customers, 62 persons (38%, sub-cohort A) scored over 21 points, whereas 101 persons (62%) scored below that number (sub-cohort B). In the group of 558 other adults, 147 persons (26%) were included into sub-cohort A, while sub-cohort B comprised of 411 persons (74%). In order to determine the factors that influenced the declaration of having above-average interest in FinTech in both cohorts, we applied the backward stepwise logistic regression model again with the dependent variable assumed as 1 when the total score for the susceptibility to use FinTech exceeded 21 (for sub-cohort A) and assumed as 0 when the score was equal to or lower than 21 (sub-cohort B). We used explanatory variables (factors) that were described in the point 2.2. of this article, i. e. which were characterising the respondents according to socio-economic features, the use of selected banking products as well as general evaluation of FinTech. The results of the estimates conducted for young customers are presented in Table 6.

**Table 6:** Determinants of the susceptibility to above-average use of FinTech offers (young customers).

	*β*	S.E.	Exp(*β*)	95% C.I. Low.	95% C.I. Up.
Use of prepaid card	1.401	0.511	4.059**	1.49	11.054
Use of credit card	0.788	0.389	2.199*	1.026	4.713
Neutral FinTech evaluation (vs. negative)	1.190	0.632	3.289*	0.953	11.353
Positive FinTech evaluation	2.398	0.639	10.998***	3.145	38.459
Constant	-2.486	0.615	0.083***		

Significance levels: *** < 0.001, ** <0.010, * <0.100.

p-value of the test of model coeff. <0.001, N = 163, α = 0.1, Cox and Snell R^2^ = 0.190, Nagelkerke R^2^ = 0.259, H-L Test = 0.392.

Source: Authors’ own calculations.

In the case of young customers, the high willingness of using FinTech was determined with three statistically significant variables. All of them concerned financial products and general evaluation of the FinTech (attitude toward FinTech).

Relevant calculations for the other adults’ cohort are presented in Table 7.

**Table 7:** Determinants of the susceptibility to above-average use of FinTech offers (adults over 30 years of age).

	*β*	S.E.	Exp(*β*)	95% C.I. Low.	95% C.I. Up.
Gymnasium Education * (vs. primary)	-23.564	28420.72	0.000	0	.
Vocational	-1.958	0.727	0.141**	0.034	0.587
Secondary	-2.121	0.724	0.12**	0.029	0.495
Bachelor	-1.317	0.839	0.268	0.052	1.387
Master	-1.326	0.736	0.265*	0.063	1.122
Ph. D.	-0.574	0.997	0.563	0.08	3.974
Non-economic education (vs. economic)	0.705	0.336	2.023*	1.047	3.908
Being in a relationship	0.568	0.298	1.765*	0.984	3.165
Independent financial decisions	0.751	0.257	2.118**	1.281	3.502
Pensioner	-1.102	0.29	0.332***	0.188	0.587
Neutral FinTech evaluation (vs. negative)	0.914	0.359	2.495*	1.233	5.047
Positive FinTech evaluation	2.79	0.37	16.276***	7.876	33.635
Constant	-1.701	0.859	0.182*		

Significance levels

*** < 0.001

** <0.010

* <0.100.

p-value of the test of model coeff. <0.001, N = 558, α = 0.1, Cox and Snell R^2^ = 0.239, Nagelkerke R^2^ = 0.349, H-L Test = 0.367.

Source: Authors’ own calculations.

In the case of the other adults’ cohort, six statistically significant variables influencing the willingness to use FinTechs beyond average were recorded. Their scope was larger than for young generation and included education, social, family, and professional situation, as well as general evaluation of the FinTech (attitude toward FinTech).

## 5. Discussion

Our research into the susceptibility to use FinTech, the experience of FinTech, and the willingness of above-average use of FinTech offers demonstrates the existence of major differences between young customers and the other adults.

Regarding the study of the susceptibility (willingness) to use the most popular FinTech products and services [32], all differences were statistically significant except for the declaration of obtaining loans through electronic platforms. The young generation average grading of likelihood of using all the seven considered FinTech products and services was higher comparing to other adults. This confirms that–when compared to the other adults–young customers generally express more interest and willingness in use FinTech offers, evaluate them better as well as are also more interested in the possibility of abandoning traditional financial products and services entirely and replacing them exclusively by FinTech.

The interest of young customers in FinTech can be explained primarily with an attractive and very modern profile of FinTechs’ operation and philosophy that–in major part–relies on mobile or online channels, which constitute an inherent part of a young person’s lifestyle, communication style, and manner of dealing with everyday matters. Thus, it is not without reason that the generations of young people are referred to as ‘digital natives’ [5,62,72]. Another explanation of why young people may find FinTech attractive is that they strongly anticipate both present and prospective users’ needs. Hardly shown by many traditional banks and other financial institutions, which must comply with numerous laws, FinTechs’ high flexibility and action dynamics also attract young customers’ attention.

Using the trend of widespread development of the digital environment FinTech may relatively easily create a broad range of products and solutions. When evaluating possibilities of FinTech development in Poland we may find that terms for digitalization and FinTech proliferation are very favourable. Such statement can be confirmed by the fact that among the 50 fastest growing technology entities classified in the Deloitte Technology Fast 50 Central Europe 2022 ranking, as many as 17 came from Poland (in 2021 it was 16) [73,74]. Poland can then be considered as the forefront, competing primarily with the Czech Republic in the scope of innovative technology companies, including the FinTech sector. FinTech companies have then conditions to create the offers and the whole market for new, attractive, innovative products and solutions which are found as interesting from the perspective of young adults.

The phenomenon of higher susceptibility to use FinTech by young adults than other adults may result also from the fact that the use of many FinTech products or services is free of charge or costs very little. For these young persons–who have a relatively low income–this is a desired situation which frequently might not happen if they wanted to use the products and services offered by traditional financial institutions that maintain physical branches.

The phenomenon of inclination of young adults toward FinTech can also be explained by the fact that in Poland there have been no spectacular bankruptcies or other types of crises related to FinTech institutions or the entire FinTech sector. Due to this reason, trust, and perception of safety of FinTech are relatively high. Moreover, young people may not necessarily familiar with the offers of banks or other financial market companies, their innovative functionalities, or digital solutions, what can result from the perception of e.g., banks, as traditional institutions associated with older generations. It should be emphasised that as young people generally do not have negative experiences with traditional financial institutions, their attitude towards FinTech is a direct result of the subjectively perceived usefulness and the ease of use, combined with benefits (low or no fees) and low risk perception. Such characteristics support the validity of the TAM theory and its variants when describing the development of the FinTech sector. Against this background, in the subgroup of other adults, less willingness in use FinTech products and solutions may be part of both their perceived lower usefulness, but also greater difficulty of use. Rapid progress in FinTech means that often other adults (especially older people) are to lesser extent able to follow change trends or learn novelties. They will be more sceptical about the usefulness and ease of use FinTech solutions. Their lower willingness to use the FinTech sector may also be influenced by the historically accumulated negative experiences in finance provided by traditional financial institutions (e.g., low quality of services, overpricing, use of prohibited clauses in contracts). Bad former experiences may distract them from searching financial innovations, that can turn harmful for them in the future. On the other hand, the lower willingness of other adults to use FinTech can be explained by the sufficient and reliable solutions provided by the traditional financial institutions.

While considering the experience of using FinTech products or services by both age cohorts that participated in the study, it must be noticed that it was possible to identify the determinants of using FinTech in respect of the respondents’ characteristics. It was observed in both cohorts that men were considerably more experienced in FinTech than women, especially in the young customers’ group. This may result from a different approach to risk adopted by men and women [75]. A similar conclusion can be drawn in respect of the household size with larger households being more likely to use FinTech. In that case, again young customers presented higher experience in FinTech than the other adults. The possibility of making financial decisions independently and the possession of a prepaid payment card also positively influenced having experience in use of FinTech in both cohorts. As with the previous cases, both determinants exert more impact on young generation’s experience of FinTech than the other adults, particularly in respect of possessing a prepaid payment card. This outcome can be compared to the results of other studies which demonstrate that the speed of transaction translates into the choice of the payment instrument [76,77], which is essential in the case of young customers [78].

Our investigation allowed also to indicate the factors determining the use and having experience in FinTech but differentiating the two study cohorts. The possession of a traditional bank account by respondents from the younger cohort cannot be overlooked as it decreases the likelihood of using FinTech. This phenomenon can be justified with the positive image, good reputation, and trust in safety of bank deposits among young people [79–81] and the fact that banks in CEE countries, especially Poland, are relatively modern and offer innovative solutions [82], owing to which they can compete with FinTech to a large extent. Regarding the latter aspect, already in 2016 Deloitte reported that Poland is the leader of digital solutions in the banking sector in CEE countries [83]. Indeed, the need for digitalization and automatization of banking operations as well as the inclination of the young adults to innovative technologies made the traditional financial institutions to become more of FinTech oriented. The more so that Fintech is perceived as one of the domains on which open banking is built [84]. Such policy led in Poland to make FinTech startups less distinctive and less differentiated from traditional banks as well as in fact turned the traditional banks into FinTech institutions with a bank licenses. Such conditions may prove that a person who has a bank account and is just gaining his or her first financial experience in an innovative bank is less susceptible to use non-bank FinTech solutions. On the other hand, the determinants of the use of FinTech among the persons over 30 years of age include the use of mobile banking and credit cards. The use of mobile banking, which is relatively modern and innovative in principle, may encourage other adult customers to reach for further, new non-banking solutions that resemble and that may complement or substitute those offered by banks. Being employed is the last factor determining the experience in use of FinTechs and concerning only the respondents over 30 years of age. In this case, the employed are more likely to use FinTech products than the persons with a different professional status.

When evaluating the high willingness to use FinTech products and services, i.e., the willingness to use it beyond average, it is important to point to the overall evaluation of FinTech operation as a statistically significant parameter characterising both age cohorts. In this case, a positive evaluation of operation influences the high willingness to use FinTech beyond average. It is worth remarking that the regression coefficient is higher for those evaluating FinTechs neutrally and lower than for those evaluating them positively in the cohort of young customers when compared to the other adults. This phenomenon implies that, regardless of how they evaluate FinTech, the persons below 30 years of age are more likely to use a FinTech beyond average than the other adults. The respondents over 30 years of age will do so on the condition that they very well evaluate FinTechs.

Regarding the determinants of the above-average use of FinTechs, which can be identified only among the young customers or only among other adults, it is important to notice among the young adults the positive impact of prepaid and credit card possession. As to the other adults, the above-average use of FinTech depends on more variables, especially those concerning being in a relationship and being able to make financial decisions independently. In this case, both parameters have a stimulating effect and may, by virtue of the respondents’ age, indicate that FinTech solutions are primarily used by the persons forming a household. Other factors influencing the widespread use of FinTech by respondents over 30 years of age include the level of education, the type of education, and the status of a retiree or a pensioner. In respect of the former, a higher level of education generally decreases the likelihood of using FinTech; however, this relationship is not linear and requires in-depth research embracing a comparison of the will of using FinTech between persons with various levels of education. We can refer here to a study by Cwynar [62] that does not confirm that Millennials differ from other generations in respect of financial literacy. Our study demonstrated that persons with non-economic education are more likely to use FinTech products and services beyond average, which, on the one hand, may result from the universality of the products and their compliance with the needs of the persons without economic education and, on the other hand, from the lack of financial expertise regarding the risks of using FinTech products and the ability to distinguish them from the offers of traditional financial institutions. The above-average use of FinTech can be observed primarily among non-retirees and non-pensioners. The professional status of a retiree or a pensioner decreases the likelihood of becoming a FinTech customer by almost 70%.

## 6. Conclusions

The purpose of the paper was to identify and evaluate the differences in the attitudes to using FinTech products and services between young adults and the other customers (other adults). Due to the fact that Poland is a post-communist country, it was assumed that the persons born no sooner than in 1990 should be recognised as young customers. The remaining adults were classified into the other cohort. The two study cohorts were born at a different time in history and were raised and came of age in a different socio-economic context.

When compared to the other adults, young customers expressed significantly more interest (higher willingness) in using non-banking FinTech in respect of all categories tested in the study, that is the susceptibility to avail oneself of seven different product areas (payments, foreign exchange, lending (non-banking institutions), lending platforms, insurance, cryptocurrencies, stock investment), the general evaluation of the operation of FinTech, and the susceptibility to use exclusively FinTech products and services. Thus, the hypothesis proposed in the paper can be confirmed.

In our research we find that majority of both age groups, i.e., young persons and other adults had experience in using non-banking FinTech solutions, with the higher rate observed among the younger generation. Such a result signifies that FinTech solutions are widespread in the economy and can be effectively used by young and older people.

Among the two groups of age there are as a rule different determinants of the use of FinTech. We found just gender, using of prepaid card, being independent in taking financial decisions and household size as the factors that turned out to be commonly stimulating to use the Fintech.

Similarly, we find different determinants of the high susceptibility (above-overage) to use FinTech products and services for both age groups studied. The determinant that turned out common for the two cohorts is the respondents’ evaluation of the FinTech. If the evaluation of the FinTech is positive, the odd of willingness to use of Fintech is clearly visible. As the other adults evaluate FinTech less positively than the young generation, this means that they are highly willing (above-overage) only if they have a positive opinion of the FinTech. This relationship is weaker for the young adults.

The influence of an age on financial behaviour and decisions should be recognised as one of the key research areas in modern finance. This conclusion stems from the observation of significant socio-demographic changes happening in the societies concurrently with rapid transformation taking place in information technology and finance.

The findings of our research undoubtedly provide practical implications which can be valuable for various commercial units. For example, thanks to our investigation, it is possible to identify the characteristics determining the use of FinTech products and services by both young and other adult customers. Financial institutions or FinTech companies might use it to increase the effectiveness of their sales. For example, the negative impact of having a bank account by young people on the willingness to use FinTech may encourage FinTechs to offer products and services to young customers before they start cooperation with traditional banks. The results of our study can be used to evaluate the possibilities of combining banking (e.g., mobile services, prepaid or credit cards) and non-banking offers with FinTech solutions as well as to develop FinTech oriented offers for customers who are actually interested in this financial segment (e.g. people who make financial decisions independently), profiled according to their age. Such an application can led to better management of customer acquisition costs, more effective support for FinTech development, as well as keeping customers loyal in terms of the increased competition that accompanies the digitalisation process.

We are conscious that our paper has a few limitations. For example, the research was conducted using the CAWI technique. Therefore, it can be assumed that the participants were the people who have free access to the Internet or mobile communication channels. The use of the CATI technique of obtaining opinions would allow to obtain opinions from people who use mobile and Internet technologies daily to the lesser extent. In addition, our study concerned the CEE market, i.e., the market where it was much easier to disseminate new financial solutions due to the lack or very low effectiveness of traditional financial solutions developed by institutions in the 90s of the twentieth century. In the Western countries, where traditional financial systems were built much earlier, the susceptibility to FinTech products and services might be different as generations of young people and adults in the Western countries did not live in such diverse (different) economic and political conditions as the societies in CEE.

The limitation of our paper was also the fact that we did not investigate the behavioural factors describing two selected age cohorts, which could be crucial from the perspective of TAM theory development. Due to the limited size of the opinion survey, we have focused mainly on socio-economic determinants.

We think that further research in intergenerational evaluation of FinTech should focus on aspects of cybersecurity and legal protection of the use of FinTech as well as aspects of the response of traditional financial institutions to FinTech activities, i.e., improving the quality of products and services, further digitisation, entering FinTech competencies. It may also be interesting to examine the impact of extraordinary situations, such as restrictions related to the COVID-19 pandemic, on the acceptance of specific technologies used in the FinTech segment.
